# Characteristics and management of tumor treating fields-related dermatological complications in patients with glioblastoma

**DOI:** 10.1097/MD.0000000000033830

**Published:** 2023-05-17

**Authors:** Chao Yang, Qing Zhang, Chao Ma, Yong Huang, Hai-Xia Ding, Jun-Wei Lu, Jie Wang, Xiang Li, Ya-Hua Zhong, Zhi-Qiang Li

**Affiliations:** a Department of Neurosurgery, Zhongnan Hospital of Wuhan University, Wuhan, China; b Department of Radiation and Medical Oncology, Zhongnan Hospital of Wuhan University, Wuhan, China.

**Keywords:** characteristics, dermatological adverse events, glioblastoma, management, tumor treating fields

## Abstract

Tumor treating fields (TTFields) is a novel approved modality for the treatment of glioblastoma (GBM) exhibiting a satisfactory effect. Although TTFields has shown considerable safety for the normal brain, dermatological adverse events (DAEs) often occur during therapy. However, studies focused on the identification and management of DAEs are rare. The clinical data and photos of skin lesions from 9 patients with GBM were retrospectively analyzed, and the types and grades of individual scalp dermatitis were evaluated based on the Common Terminology Criteria for Adverse Events version 5.0 (CTCAE v 5.0). Adherence and safety were also evaluated on the basis of the device monitoring data. Eight patients (88.9%) exhibited grade 1 or grade 2 CTCAE DAEs, all of whom were cured after interventions. The adherence was >90%, with no relevant safety events reported. Finally, a guideline for preventing DAEs in patients with GBM was proposed. The identification and management of TTFields-related DAEs is necessary and urgent in patients with GBM. Timely interventions of DAEs will help to improve the adherence and quality of life of patients, which ultimately improves prognosis. The proposed guideline for preventing DAEs in patients with GBM assists in the management of healthcare providers and may avoid dermatologic complications.

## 1. Introduction

Glioblastoma (GBM) is the most common malignant brain tumor in adults and has a poor prognosis despite many great advances in diagnosis and treatment.^[1]^ The standard Stupp protocol for patients with GBM includes maximum safe resection, followed by concurrent chemoradiotherapy and adjuvant temozolomide (TMZ)^[2]^; however, the median overall survival (OS) of patients with GBM is currently only 15 months. Moreover, almost all patients with GBM experience disease recurrence due to unclear tumor margins and limited efficacy of chemoradiotherapy. Advances in sequencing technology have revealed high tumor heterogeneity,^[3]^ while the blood–brain barrier is known to limit the entry of systematic drugs,^[4]^ partially contributing to problems with traditional therapies.

The biological effects of low-density, intermediate frequencies (100–300 kHz) alternating electric fields have been reported in recent decades. Tumor Treating Fields (TTFields; Optune, Novocure Inc, NJ) is a novel physical therapy device that involves attaching accurate transducer arrays to the patients’ shaved scalp and delivering low-density, alternating electric fields (200 kHz) to the tumor.^[5]^ TTFields cause the death of tumor cells by interfering with the formation of mitotic spindle microtubules^[5,6]^ and can induce tumor cell apoptosis,^[7]^ DNA replication stress,^[8]^ and immunogenic cell death,^[9]^ among others. TTFields was first approved for clinical application in 2009 in the European Union and subsequently gained approval for clinical application in the USA and Japan. In 2020, the clinical application of TTFields was also approved by the National Medical Products Administration for both recurrent and newly diagnosed GBM. Two previous phase 3 clinical trials identified the value of TTFields in GBM; among which, the EF-11 trial,^[10]^ focusing on a cohort with recurrent GBM, failed to demonstrate superiority compared with active chemotherapy alone, with a median OS of 6.6 versus 6.0 months, respectively. Intriguingly, in another EF-14 trial on patients with newly diagnosed GBM,^[11]^ significant improvements in OS were observed between the combination of TTFields and TMZ and TMZ alone, with a median OS of 20.9 versus 16.0 months, respectively. Independent prognostic significance of increased compliance with TTFields therapy was also reported, and patients with >90% compliance showed extended median and 5-year survival rates.^[12]^

Compared with the frequent hematologic, gastrointestinal, and infection adverse events associated with traditional chemoradiotherapy, the most common adverse events of TTFields are dermatologic adverse events (DAEs).^[13,14]^ Indeed, in the EF-14 trial, 52% of patients in the combined TTFields and TMZ group had DAEs, all of which were Common Terminology Criteria for Adverse Events (CTCAE) grade 1 or grade 2. Importantly, the addition of TTFields did not increase the incidence of other systemic adverse events.^[11]^ Furthermore, 16% of patients with recurrent GBM receiving TTFields were reported to have grade 1 or grade 2 DAEs, and all were manageable and reversible.^[10]^ Although the CTCAE has previously described TTFields-related DAEs in clinical trials in GBM,^[15]^ some TTFields-related specific manifestations could not be described and graded accurately. Recent studies have attempted to clarify the characteristics and management of DAEs in patients with GBM receiving TTFields,^[16]^ while the current study focuses on the lack of a standard management strategy for TTFields-related DAEs. Thus, a suitable evaluation and management system of DAEs is required for patients with GBM receiving TTFields.

In this study, we systematically characterized the type and severity of DAEs in patients with GBM receiving TTFields in our hospital and proposed a series of preventive strategies against scalp damage during TTFields. Our results will help oncologists and healthcare providers to improve the identification and treatment of TTFields-related DAEs in patients with GBM.

## 2. Material and Methods

### 2.1. Study cohort

Nine adult patients who received TTFields and were diagnosed with GBM in the Department of Neurosurgery at the Zhongnan Hospital of Wuhan University from August 2020 to August 2021 were retrospectively incorporated. The inclusion criteria were as follows: age ≥ 18 years; surgery or biopsy was performed and the diagnosis was histologically confirmed based on the 2016 World Health Organization classification of tumors of the central nervous system; and clinical information, image data of DAEs, and usage details of the device of patients receiving TTFields were available. The Exclusion criteria were as follows: prior history of cancer and short duration of TTFields (≤1 course). All procedures followed the Helsinki Declaration.^[17]^ The last follow-up was conducted on December 31, 2021. This study followed the STROBE checklist.

### 2.2. Ethics approval

This study was approved by the Ethics Committee of Zhongnan Hospital of Wuhan University (No. 2019048).

### 2.3. Data collection

Demographic and clinical information was obtained from an electronic medical system and included sex, age at diagnosis, pretreatment Karnofsky performance status, types of GBM, phase of using TTFields, extent of resection (gross total resection [GTR] ≥ 95%, non-GTR < 95%), mutation of *isocitrate dehydrogenase 1*, methylation of the O^6^-methylguanine-DNA methyltransferase promoter, and statuses of codeletion of chromosome 1p and 19q. The image data of the scalps of patients were derived from photos taken by a medical worker in the hospital or healthcare providers at home. The analysis of adherence and safety was based on feedback data from the TTFields modality. Evaluations of the types, severity, and grade of DAEs in patients with GBM who received TTFields were subjected to CTCAE v5.0.

### 2.4. Statistical analysis

The normal distribution of continuous variables is expressed as the mean ± standard deviation, and the non-normal distribution is expressed as the median and interquartile range (IQR) and was analyzed by nonparametric tests or *t* tests. Categorical variables are described as frequencies (percentages) and were compared between groups using chi-square test. Statistical analysis was performed using SPSS (version 24.0, IBM Corporation, Armonk, NY).

## 3. Results

### 3.1. Clinical characteristics

The clinical characteristics of all patients are presented in Table 1. Nine patients (one female and 8 males) were included in our study, including 8 with primary GBM and one with secondary GBM. The mean age of all patients was 48.7 years (range: 20–74 years) and the mean Karnofsky Performance Status was 60 (range: 30–100). Among all patients, 33.3% underwent GTR, 55.6% underwent non-GTR, and 11.1% patient received biopsy only. All patients received TTFields during concurrent chemoradiotherapy and did not harbor codeletion of chromosomes 1p and 19q. One (11.1%) and 4 (44.4%) patients with GBM harbored an *isocitrate dehydrogenase1* R132H mutation or O^6^-methylguanine-DNA methyltransferase promoter methylation, respectively. The composition and wearing of the TTFields device are shown in Supplementary Figure 1, http://links.lww.com/MD/J14.

**Table 1:** Clinical and pathological characteristics of patients with GBM receiving TTFields.

Characteristics	All patients
Count, n (%)	9 (100)
Sex, n (%)	
Female	1 (11.1)
Male	8 (88.9)
Age (yr) (mean ± SD)	48.7 ± 16.6
KPS (mean ± SD)	60.0 ± 31.2
Primary GBM, n (%)	
Yes	8 (88.9)
No	1 (11.1)
Phase using TTFields	
Concurrent chemoradiotherapy	9 (100)
Resection, n (%)	
GTR	3 (33.3)
Non-GTR	5 (55.6)
Biopsy	1 (11.1)
*IDH1*^R132H^, n (%)	
Yes	1 (11.1)
No	8 (88.9)
*MGMT* promoter methylation, n (%)	
Yes	4 (44.4)
No	5 (55.6)
Codeletion of chromosome 1p and 19q	
No	9 (100)

KPS = Karnofsky performance status, GBM = glioblastoma, GTR = gross total resection, IDH = isocitrate dehydrogenase*, MGMT* = O^6^-methylguanine-DNA methyltransferase, TTFields = tumor treating fields.

### 3.2. Analysis of adherence and safety

**Figure 1.**
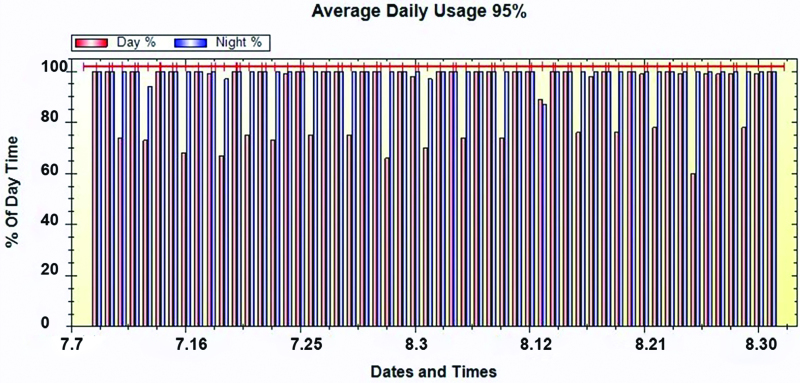
Adherence and safety analysis in a patient with GBM receiving TTFields. GBM = glioblastoma, TTFields = tumor treating fields.

The device usage details were downloaded, as shown in Figure 1, which describes the frequency of usage of a patient from July 7 to August 30 in 2021. On average, the adherence was >90%, and the safety of the device was guaranteed if the temperature was >41ºC through the preset temperature sensors. During the TTFields treatment, we continually monitored the progression of DAEs, which may have been caused by the installation of the device.

### 3.3. Types, grade, and possible causes of DAEs in patients with GBM receiving TTFields

**Figure 2.**
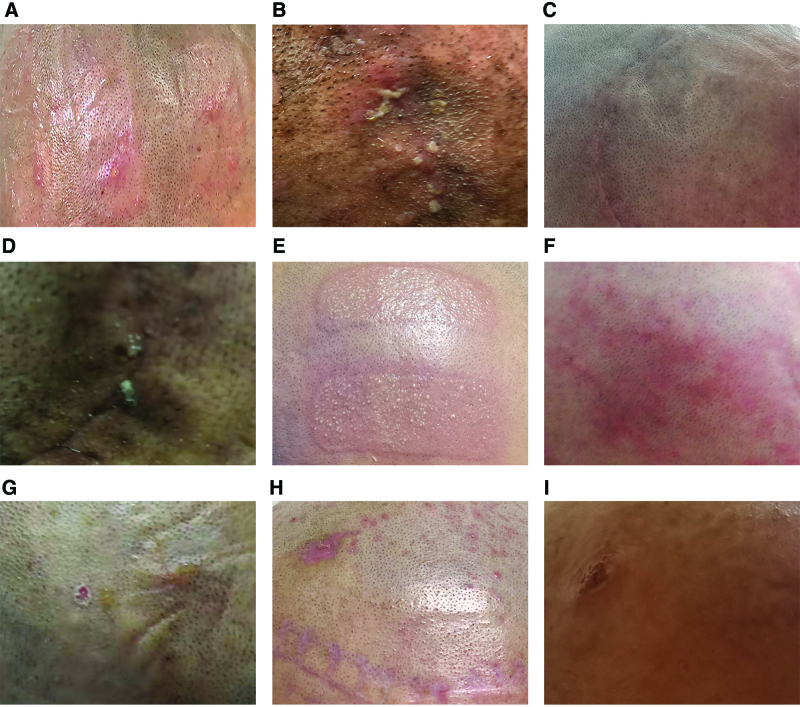
Type, severity, and grade of dermatological adverse events in patients with GBM receiving TTFields. Cases A–I represent the corresponding individual. GBM = glioblastoma, TTFields = tumor treating fields.

An evaluation of the type, severity, and grade of DAEs of all patients was performed according to the CTCAE v5.0 (Fig. 2 and Table 2). Four patients developed contact irritant or allergic dermatitis beneath the transducer arrays (Fig. 2A, E, F, H). Potential causes of contact irritant dermatitis may be a type of nonspecific inflammation caused by direct contact of some irritant substance, such as hydrogel, moisture, and alcohol, with scalp epidermal cells. Contact allergic dermatitis was likely caused by an allergic reaction to exogenous allergens, such as adhesive tapes or hydrogel. Infection occurred in 2 patients (Fig. 2B and D) because of secondary bacterial infection on the scalp. Erosion was observed in 2 patients (Fig. 2G and I) and may have been caused by mechanical injury from shaving and/or application or removal of the transducer array. One patient had no DAEs during TTFields therapy (Fig. 2C). All 8 patients were graded as having level 1/2 dermatitis according to the CTCAE guidelines (Table 2).

**Table 2:** Characteristics of dermatology AEs in patients with GBM receiving TTFields.

Case	Types of dermatology AEs	CTCAE grade	Reasons	Treatment	Outcome
1	Contact dermatitis	1	Allergic to specific allergens and/or direct contact with chemical irritation	Topical antibiotic (mupirocin) and application of a barrier film (aloe vera gel)	Recovery
2	Dermatitis + infection	2	Secondary bacterial infection	Topical antibiotic (mupirocin)	Recovery
3	Normal	/	/	/	/
4	Dermatitis + infection	2	Secondary bacterial infection	Topical antibiotic (mupirocin)	Recovery
5	Contact dermatitis	2	Allergic to specific allergens and/or direct contact with chemical irritation	Removal of transducer arrays and interruption of treatment	Recovery
6	Contact dermatitis	2	Allergic to specific allergens and/or direct contact with chemical irritation	Topical antibiotic (mupirocin) and application of a barrier film (aloe vera gel)	Recovery
7	Erosion	2	Mechanical injury or development from inflammation	Topical antibiotic (mupirocin)	Recovery
8	Contact dermatitis	1	Allergic to specific allergens and/or direct contact with chemical irritation	Topical camphor cream	Recovery
9	Erosion	2	Mechanical injury or development from inflammation	Topical antibiotic (mupirocin)	Recovery

Cases 1–9 represent the corresponding patient.

CTCAE = common terminology criteria for adverse events, GBM = glioblastoma, TTFields = tumor treating fields.

### 3.4. Interventions for the DAEs in patients with GBM receiving TTFields

**Figure 3.**
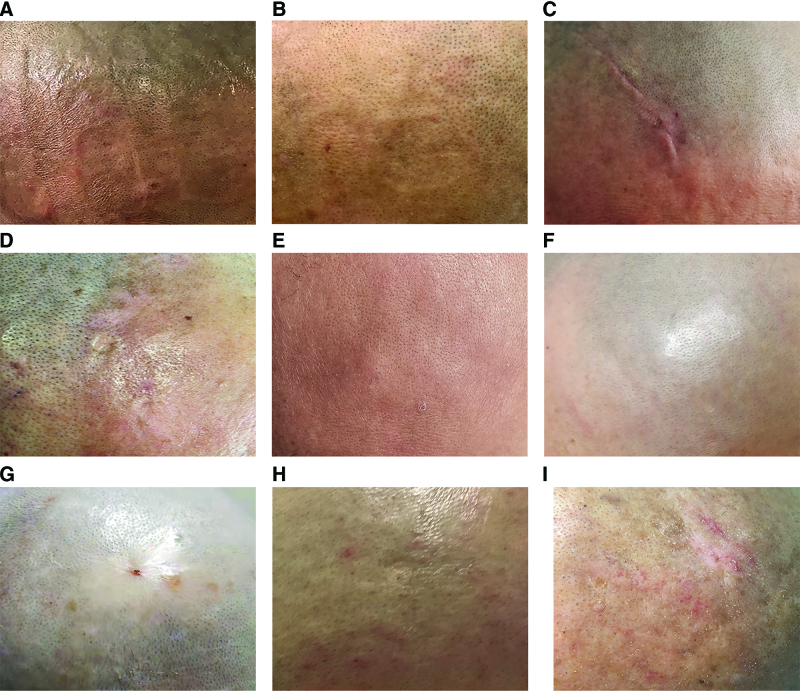
Post-treatment DAEs in GBM patients receiving TTFields. Case A-I represents the corresponding individual. DAEs = dermatological adverse events, GBM = glioblastoma, TTFields = tumor treating fields.

The interventions for DAEs were applied based on the different types of DAEs and considering different possible pathological mechanisms (Table 2). Many measures could be taken to cure contact dermatitis, including immediate removal of any potential irritant/allergen and transducer arrays, topical corticosteroids (systemic use of corticosteroids if the lesion is persistent, although a break from therapy would be necessary). For treating infection, a topical antibiotic is an effective choice, and an oral/systemic antibiotic or a break from therapy would be required if the lesion became aggravated. For the erosion of scalp cells, removal of transducer arrays, dressing with gauze and/or hydrogel, and antibiotic usage may be needed. All 8 patients recovered from their DAEs after timely and appropriate interventions (Fig. 3).

### 3.5. Proposed prophylactic interventions of DAEs in patients with GBM receiving TTFields

Long-term contact between the transducer arrays and scalp inevitably increased the risk of DAEs in patients with GBM. Considering that the usage time is significantly correlated with the outcome in patients with GBM, it is crucial to identify potential risk factors and avoid DAEs as far as possible. A series of preventive strategies are proposed based on previous literature and clinical experience (Table 3). Before the application of TTFields, feasible methods consist of identification of risk factors, preparation of the scalp, application of transducer arrays, and further education of health managers. During and after therapy, regular and careful changes of transducer arrays, further education, and timely cleaning of the scalp were effective measures to reduce the risk of DAE.

**Table 3:** Proposed prophylactic interventions of DAEs in patients with GBM receiving TTFields.

Pretreatment		During and post-treatment	
Identification of risk factors	Surgery scars and hardware; Hyperhidrosis; History of dermatitis; Persistent alopecia; History of radiation exposure; Allergic constitution	Transducer arrays	Careful regular changes; Careful removal of arrays
Preparation of scalp	Optimal shaving using a clean electric razor; Removal of natural oils and sweat	Education	Improving the ability to identify dermatitis; Avoidance of the application of arrays directly to scar lines or surgery hardware
Transducer arrays	Careful application	Cleanness of the scalp	Timely cleanness of the scalp after removal of transducer arrays
Education	Education of patients and care providers		

DAEs = dermatological adverse events, GBM = glioblastoma, TTFields = tumor treating fields.

## 4. Discussion

The poor prognosis of GBM has inspired oncologists to explore the biological behavior of the tumor and identify additional effective therapy modalities besides maximum safe resection and chemoradiotherapy. TTFields is a novel, noninvasive, and safe physiotherapy, which has been approved for clinical application in patients with primary and recurrent GBM, and has shown significant improvement in OS and relatively mild-to-moderate adverse events. DAEs are the most common adverse events during TTFields. In this study, the characteristics and corresponding treatments of DAEs observed in 9 patients with GBM in our glioma center were reported (Fig. 3). All of the DAEs were grade 1 or grade 2 based on CACTE v5.0, and all recovered completely after timely intervention, accompanied by a high safety and adherence of patients. The proposed effective prophylactic plan for DAEs in the peri treatment of TTFields will be important for the management of routine clinical practice in the future.

In China, TTFields was first approved for use in clinical practice in 2020. TTFields involves attaching 2 orthogonally arranged transducer arrays to the shaved scalps of patients with primary and recurrent GBM.^[18]^ Although electric fields (different frequencies and intensities) have been proven to have significant effective biological effects and have been available in the clinic for many years, alternating electric fields at intermediate frequencies (100–300 kHz) and low intensity (1–3 V/cm) did not gain attention until recent decades. TTFields uses alternating electric fields with intermediate frequencies and low intensity and has the potential to suppress tumor initiation, development, and metastasis. Two previous clinical trials accelerated the clinical application of TTFields in GBM; the EF-11 trial, focusing on cohorts with recurrent GBM, compared TTFields with active chemotherapy including a single agent or a combination of bevacizumab, carboplatin, and TMZ. The results showed that the OS and progression-free survival were comparable in the 2 groups, with no significant difference. However, a significantly better outcome was observed in the TTFields group compared with the TMZ-alone group in another EF-14 clinical trial in newly diagnosed patients with GBM. Besides, electric fields have also been administered in many other solid tumors and have achieved satisfactory effects.^[19,20]^

The potential mechanisms behind TTFields have been widely studied, and anti-mitotic effects have also been demonstrated by interfering with the formation of mitotic spindle microtubules, with no effect on non-mitotically active cells.^[5,6]^ Other possible mechanisms involve autophagy upregulation,^[21]^ DNA repair inhibition,^[8]^ and angiogenesis suppression.^[9]^ A previous study reported that TTFields could increase the permeability of tumor cells and the blood–brain barrier, which may help to improve the effect of systemic agents, while many current trials are focusing on the combination of TTFields and traditional chemotherapy agents. The safety of the device was also guaranteed by 8 temperature sensors that were preset to each array to monitor the temperature in real time; in the event that the device exceeded 41ºC, it would shut off automatically and sound an alarm.^[22]^

In previous clinical trials, DAEs have been shown to be the most common adverse events during TTFields therapy, although most were mild and moderate skin complications with a reversible outcome. In our study, 8 patients developed grade 1/2 DAEs and all recovered completely after related interventions. The skin is composed of 3 layers, including the epidermis, dermis, and hypodermis, as well as the associated subcutaneous fat.^[23]^ Most treatment-related dermatologic injuries are located in the epidermis layer, while severe infection and ulcers may invade the second and third layers. A previous study^[16]^ identified 5 types of DAEs in patients with GBM receiving TTFields, including hyperhidrosis, pruritus, contact dermatitis, erosion and ulcer, and infections. It will be more accurate and effective to treat and prevent DAEs based on their different types and mechanisms. According to the EF-14 trial, patients with GBM should use the device for 18 hours a day on average,^[11]^ and the use time of TTFields has been shown to be an independent prognostic factor in patients with GBM.^[12,24]^ However, few studies have reported the standard treatment for DAEs in GBM, and a current accurate classification system focused specifically on TTFields-related DAEs is lacking because the existing CTCAE classification system cannot describe some characteristic skin changes.

This study has several limitations that warrant discussion. First, the retrospective nature of our study may have led to selection bias. Second, as a relatively small number of patients with GBM receiving TTFields were included, our results do not comprehensively describe all types of DAEs. Hence, future large-scale and prospective studies are needed to clarify these concerns.

## 5. Conclusions

The identification and management of TTFields-related DAEs is both necessary and urgent in patients with GBM. Timely interventions of DAEs help to improve patient adherence and quality of life, which ultimately improves prognosis. The proposed guideline for preventing DAEs in patients with GBM will help to improve the management of healthcare providers and avoid dermatologic complications.

## Acknowledgments

We thank LetPub (www.letpub.com) for its linguistic assistance during the preparation of this manuscript and thank Ji-Quan Song for his help in the identification of types of DAEs. We thank Professor Yi Guo for his help in the procedure of statistical analysis.

## Author contributions

**Conceptualization:** Zhi-Qiang Li.

**Data curation:** Chao Yang, Qing Zhang, Jun-Wei Lu, Jie Wang.

**Formal analysis:** Chao Yang, Hai-Xia Ding.

**Investigation:** Chao Ma.

**Methodology:** Yong Huang.

**Supervision:** Xiang Li, Zhi-Qiang Li.

**Writing – original draft:** Chao Yang.

**Writing – review & editing:** Ya-Hua Zhong, Zhi-Qiang Li.

## Supplementary Material
