# Supplementary material for: Characteristics and management of tumor treating fields-related dermatological complications in patients with glioblastoma
Source: Medicine (Baltimore). 2023 May 17;102(20):e33830. doi: 10.1097/MD.0000000000033830 (PMC10194479; doi:10.1097/MD.0000000000033830)
Supplement: Supplementary file 1 [file medi-102-e33830-s001.pdf]

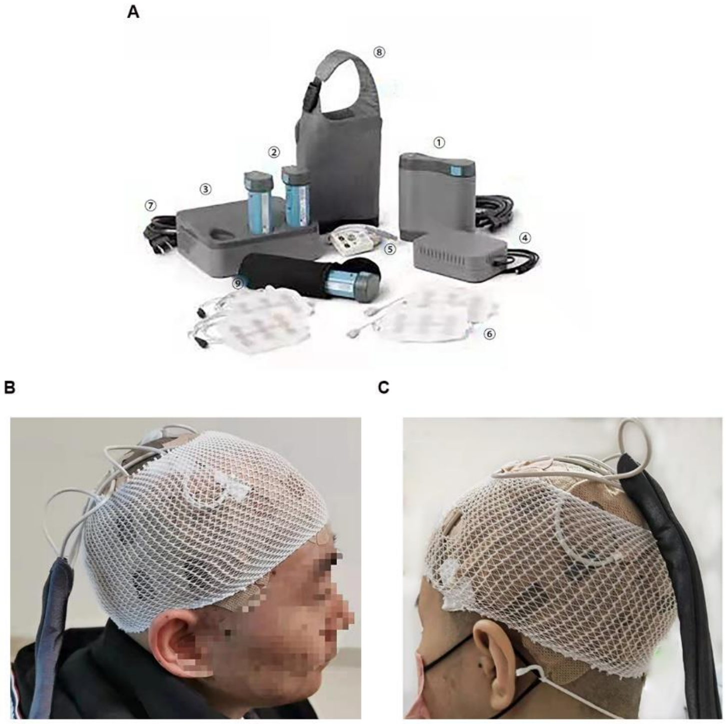

Supplementary Figure 1 Composition and wearing of the TTFields device. **A.** Composition of the TTFields device: ① Field generator device; ② Portable battery; ③ Battery charger; ④ Plug-in power supply; ⑤ Connection cables and box; ⑥ Transducer arrays; ⑦ Power line; ⑧ Shoulder bag; ⑨ Portable battery pack. **B and C.** Wearing of the TTFields device
